# A Broad Overview of Signaling in *Ph*-Negative Classic Myeloproliferative Neoplasms

**DOI:** 10.3390/cancers13050984

**Published:** 2021-02-26

**Authors:** Ana Guijarro-Hernández, José Luis Vizmanos

**Affiliations:** 1 Department of Biochemistry and Genetics, School of Sciences, University of Navarra, 31008 Pamplona, Spain; aguijarro@unav.es; 2 Navarra Institute for Health Research (IdiSNA), 31008 Pamplona, Spain

**Keywords:** myeloproliferative neoplasms, signaling pathways, JAK2, CALR, MPL, TPOR

## Abstract

**Simple Summary:**

There is growing evidence that *Ph*-negative myeloproliferative neoplasms are disorders in which multiple signaling pathways are significantly disturbed. The heterogeneous phenotypes observed among patients have highlighted the importance of having a comprehensive knowledge of the molecular mechanisms behind these diseases. This review aims to show a broad overview of the signaling involved in myeloproliferative neoplasms (MPNs) and other processes that can modify them, which could be helpful to better understand these diseases and develop more effective targeted treatments.

**Abstract:**

*Ph*-negative myeloproliferative neoplasms (polycythemia vera (PV), essential thrombocythemia (ET) and primary myelofibrosis (PMF)) are infrequent blood cancers characterized by signaling aberrations. Shortly after the discovery of the somatic mutations in JAK2, MPL, and CALR that cause these diseases, researchers extensively studied the aberrant functions of their mutant products. In all three cases, the main pathogenic mechanism appears to be the constitutive activation of JAK2/STAT signaling and JAK2-related pathways (MAPK/ERK, PI3K/AKT). However, some other non-canonical aberrant mechanisms derived from mutant JAK2 and CALR have also been described. Moreover, additional somatic mutations have been identified in other genes that affect epigenetic regulation, tumor suppression, transcription regulation, splicing and other signaling pathways, leading to the modification of some disease features and adding a layer of complexity to their molecular pathogenesis. All of these factors have highlighted the wide variety of cellular processes and pathways involved in the pathogenesis of MPNs. This review presents an overview of the complex signaling behind these diseases which could explain, at least in part, their phenotypic heterogeneity.

## 1. Introduction

Myeloproliferative neoplasms (MPNs) are rare hematological malignancies characterized by the clonal expansion of mature myeloid cells. MPNs arise from certain somatic mutations in hematopoietic stem cells (HSCs) which provide a selective advantage and lead to the expansion of aberrant clones.

Classic MPNs consist of chronic myeloid leukemia (CML), polycythemia vera (PV), essential thrombocythemia (ET) and primary myelofibrosis (PMF). In the last few years, the advances in molecular biology have provided key insights into the molecular mechanisms behind these diseases. CML is genetically defined by the *Philadelphia* (*Ph*) chromosome, the result of t(9;22)(q34;q11). This translocation leads to the production of a chimeric BCR-ABL1 protein with constitutive tyrosine kinase activity. The description of the *Ph* chromosome as a disease-initiating event in CML revolutionized the diagnosis and treatment of this disease [1]. The targeted therapy imatinib showed a specific inhibitory capacity against the tyrosine kinase activity of BCR-ABL1 [2,3,4] that, despite not being curative [5], increased the 10-year survival of CML patients in chronic phase to more than 83%–84% [6,7].

This review is focused on PV, ET and PMF, all of them *Ph*-negative MPNs that share similar and mostly mutually exclusive driver mutations affecting *JAK2*, *MPL* and *CALR*. The aberrant functions of the mutant products encoded by these genes have been extensively studied and the main mechanisms that lead to the myeloproliferation described. Currently, it is considered that the major hallmark of *Ph*-negative MPNs is the constitutive activation of JAK2-related signaling pathways. In fact, at this time, the only targeted therapy approved in MPNs is the JAK1/2 inhibitor ruxolitinib, which can reduce splenomegaly and other common symptoms in patients with PMF, post-PV/ET MF [8,9] and PV resistant or intolerant to hydroxyurea [9,10]. Although a reduction in the mutant allele burden is rare [9], it could be achieved in long-term treatment [11]. However, the improvement in the overall survival of ruxolitinib-treated patients has been questioned [12,13,14]. Actually, malignant cells can still survive in these patients and the clinical response could be mainly due to the downmodulation of proinflammatory cytokines derived from the JAK2 inhibition [15]. These arguments have led researchers to question whether JAK2 is really the best drug target in these diseases or not [16].

In the meantime, some non-canonical mechanisms of mutant JAK2 [17,18,19,20,21,22,23,24] and CALR [25,26,27,28,29,30,31,32,33] have been described. Chronic inflammation [34,35,36,37,38,39,40,41,42,43,44,45,46,47,48,49,50,51,52,53,54,55,56,57,58,59,60,61,62] and the bone marrow microenvironment [63,64,65,66,67,68,69,70,71,72] also seem to contribute to the heterogeneous phenotypes found among MPN patients.

Additionally, mutations in disease-modifying genes that seem to increase the risk of leukemic transformation or progression from ET to myelofibrosis have also been identified [73,74,75]. The products encoded by these genes are involved in epigenetic modification, tumor suppression, transcription regulation, splicing, and some other signaling pathways [76,77]. Other factors, such as genetic predisposition, age or environment have also been shown to influence the heterogeneity of MPN phenotypes [78].

This review presents an overview of the signaling behind *Ph*-negative MPNs attending not only to the activation of JAK2-related canonical signaling pathways, but also to other non-canonical pathways, disease-modifying signaling, and additional factors that have been found to be involved in the pathogenesis of these diseases.

## 2. JAK2-Related Canonical Signaling Pathways

JAK2 signaling is activated through a variety of receptors such as those for erythropoietin (EPOR), thrombopoietin (TPOR), and granulocyte/macrophage colony-stimulating factor (GM-CSFR). They regulate the production of the erythroid, megakaryocytic, and granulocytic lineages, respectively. When stimulated by ligands, receptors dimerize and bring JAK2 kinases into proximity. JAK2 is phosphorylated upon receptor binding and induces the phosphorylation of the cytoplasmic portion of the receptor and downstream factors.

**Figure 1:**
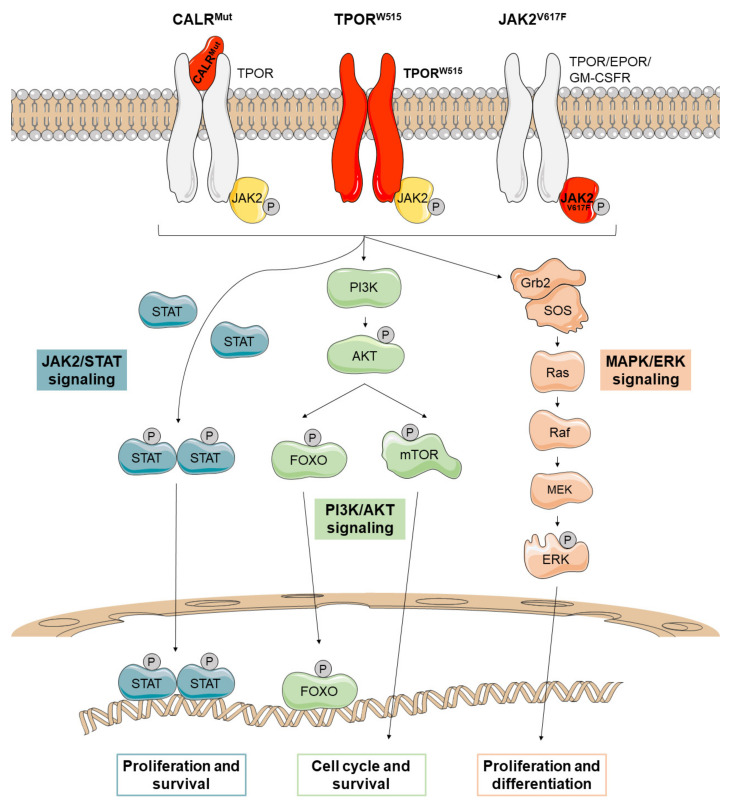
JAK2-related canonical signaling pathways active in *Ph*-negative myeloproliferative neoplasms (MPNs). Mutations in *CALR* (CALR^MUT^), *JAK2* (JAK2^V617F^), and *MPL* (TPOR^W515^) lead to the constitutive activation of JAK2/STAT, PI3K/AKT, and MAPK/ERK signaling that promotes the transport to the nucleus of several transcription factors such as STATs and FOXO. There, they regulate transcription of their target genes, causing increased proliferation and survival of mutant cells. Mutant proteins are depicted in red.

In 2005, several research groups simultaneously published the presence of the somatic mutation p.V617F (JAK2^V617F^) in the exon 14 of *JAK2* in patients with PV (96%), PMF (65%) and ET (55%) [79,80,81,82,83,84]. This mutation impairs the physiological inhibitory function of the JH2 pseudokinase domain upon the JH1 kinase domain, which acquires a constitutive activation that promotes JAK2 phosphorylation in the absence of ligand stimulation (Figure 1). In 2007, four additional gain-of-function somatic mutations in the exon 12 of *JAK2* were detected in 3% of patients with PV [84,85]: p.N542-E543del (30%), p.K539L (14%), p.E543-D544del (12%), and p.F537-K539delinsL (10%). All of them are located upstream of the JH2 pseudokinase domain and promote an increased phosphorylation of JAK2 compared to p.V617F [86].

In 2006, the gain-of-function mutation p.W515L in the exon 10 of *MPL* was identified in a minor proportion of MPN patients [87]. p.W515L and p.W515K are the most commonly reported mutations, identified in approximately 5% of PMF patients and 1% of ET patients [88]. *MPL* encodes the thrombopoietin receptor (TPOR), which depends on JAKs to mediate signal transduction. *MPL* mutations (TPOR^W515^) promote the dimerization and activation of TPOR, leading to transphosphorylation and activation of the previously bound JAK2 proteins (Figure 1) [89].

The molecular alteration that causes the 60–90% of PMF and ET cases in patients not harboring *JAK2*/*MPL* mutations was described in 2013 [90]. During that year, two research groups identified mutations in *CALR* [91,92], a gene that encodes calreticulin, a ubiquitous protein found in the endoplasmic reticulum (ER) of all nucleated cells with multiple functions inside and outside this organelle. CALR is a Ca^2+^-binding chaperone mainly involved in the regulation of intracellular Ca^2+^ homeostasis and a regulator of protein folding in the cellular response to ER stress (unfolding protein response (UPR)) [93]. However, this protein has been also found associated with other cytoplasmic, nuclear and extracellular proteins, so it could be involved in a wide variety of signaling pathways [94]. In fact, CALR has been associated with cellular stress responses, adipocyte differentiation, cardiogenesis, proliferation, wound healing, apoptosis and immunogenic cell death [90,94].

The structure of wild-type CALR consists of a signal peptide and three domains: an amino-terminal N-domain, a proline-rich P-domain and a carboxy-terminal C-domain, which contains an ER retention signal (KDEL). The *CALR* mutations described to date are insertions or deletions in exon 9 that shift the reading frame by one base pair (+1), mainly a 52-bp deletion (c.1902_1143del) or type 1 mutation (CALR^del52^), and a 5-bp insertion (c.1154_1155insTTGTC) or type 2 mutation (CALR^ins5^). As a result, mutant CALRs (CALR^Mut^) show a novel C-terminal end that lacks the ER retention motif (KDEL) [91,92] and some Ca^2+^-binding sites [95]. In 2016, it was published that CALR^Mut^ is transported to the cellular membrane where it activates TPOR in a ligand-independent manner (Figure 1) [96,97,98,99]. The characterization of the TPOR binding capacity has revealed that the C-terminal end of CALR^Mut^ blocks the P-domain of the protein, which constitutively exerts an inhibitory effect on the N-domain. Consequently, the N-domain can bind to immature N-glycans on TPOR [96]. This mechanism is consistent with the observation that the N-glycan binding motif located in N-domain of CALR^Mut^ is required for TPOR activation [97]. In fact, blocking N-glycosylation on asparagine 117 of TPOR diminishes CALR-dependent TPOR activation [97,100]. Both wild-type and mutant CALR recognize immature forms of N-glycans and fold the protein correctly, but CALR^Mut^ fails to dissociate from the targeted protein [101]. Thus, the CALR^Mut^-TPOR complex moves from the ER to the plasma membrane through the Golgi apparatus and is secreted out of cells [102]. However, secreted CALR^Mut^ is only capable to activate TPOR on the cell surface of cells expressing CALR^Mut^ since only these cells have the immature N-glycans on TPOR [96,102,103]. Stimulation of TPOR leads to the activation of JAK2-dependent signaling in a similar way to the rest of the *Ph*-negative MPNs.

In conclusion, the mutations described to date in *JAK2*, *MPL* and *CALR* lead to a constitutive activation of JAK2, which ultimately causes the aberrant proliferation and survival of malignant myeloid clones. The three major downstream signaling pathways that are activated by JAK2 are JAK2/STATs, MAPK/ERK, and PI3K/AKT (Figure 1). The evidence suggests that each of these pathways plays an important role in MPNs, although the JAK2/STAT pathway appears to be the main one. In fact, dysregulation of JAK2/STAT signaling has been identified in all MPNs regardless of mutational status [104].

### 2.1. JAK2/STAT Pathway

In MPNs, JAK2 phosphorylates and activates STATs (mainly STAT1, STAT3 and STAT5). It seems that STATs are differentially activated depending on the type of MPN. For example, *MPL* mutations increase STAT3 and STAT5 signaling. In PV patients, JAK2^V617F^ binds to EPOR promoting STAT5 activation. In ET patients, both JAK2^V617F^ and CALR^Mut^ bind to TPOR; JAK2^V617F^ enhances the phosphorylation of STAT1 and STAT3 but CALR^Mut^ promotes STAT3 and STAT5 activation. However, in PMF, phosphorylation of STAT3 is decreased in both JAK2^V617F^ and mutant CALRs. To date, the precise mechanisms that explain differential activation of the STATs remain unclear [78].

Once the STATs are phosphorylated, they form a dimer that enters the nucleus to activate the transcription of target genes (Figure 1). In this way, JAK2/STAT signaling stimulates cell proliferation, differentiation and survival.

### 2.2. MAPK/ERK Pathway

The activated JAK2 can also lead to the phosphorylation of ERK, a serine threonine kinase that activates multiple proteins in both the cytoplasm and the nucleus. ERK is a key regulator of a wide variety of signaling pathways, so its deregulation could disrupt multiple processes. In the cytoplasm, ERK contributes to ion transport, apoptosis, and regulation of metabolism, among others. In the nucleus, it targets regulators of cell cycle and multiple transcription factors (Figure 1) [105].

### 2.3. PI3K/AKT Pathway

JAK2 activation also stimulates the PI3K/AKT pathway. AKT is a cell survival kinase which inhibits apoptosis by phosphorylating the proapoptotic protein BAD and the transcription factor FOXO3A. In addition, AKT can activate a wide range of mechanisms such as protein translation through mTOR or the cell cycle machinery (Figure 1) [105].

## 3. Non-Canonical Signaling Pathways

### 3.1. JAK2-Related Non-Canonical Signaling

**Figure 2:**
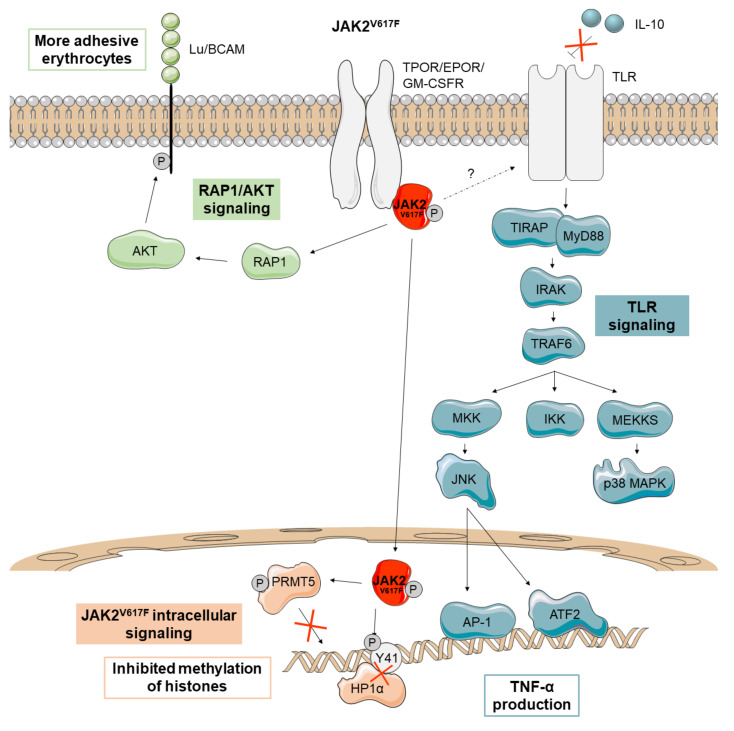
Main non-canonical signaling pathways activated by JAK2^V617F^ in *Ph*-negative MPNs. In PV patients, JAK2^V617F^ (depicted in red) activates the adhesion receptor Lu/BCAM through the RAP1-AKT signaling pathway, making their erythrocytes more adhesive. JAK2^V617F^ has also been described to promote aberrant signaling in the nucleus, where it prevents the binding of heterochromatin protein 1 alpha (HP1α) and inhibits the methylation of histones via protein arginine methyltransferase 5 (PRMT5) impairment. MPN patients with JAK2^V617F^ also seem to be insensitive to the anti-inflammatory cytokine IL-10, increasing TNF-α production through Toll-Like Receptor (TLR) signaling.

In 2009, activated JAK2 was described to be in the nucleus of hematopoietic cells and to phosphorylate Y41 on histone 3 (H3Y41). This event prevents the binding of heterochromatin protein 1 alpha (HP1α) to H3Y41 [17]. HP1α shows a proliferation-dependent regulation and is involved in gene silencing, genome stability, and chromosome segregation (Figure 2). It has been found overexpressed in some tumors, and it has been proposed as a potential hallmark of cell proliferation that could be relevant in clinical oncology [18].

JAK2^V617F^ also acquires the ability to phosphorylate the protein arginine methyltransferase 5 (PRMT5) leading to an impairment in its ability to methylate histones (Figure 2). When PRMT5 is knocked down in CD34+ cells, an increased colony formation and erythroid differentiation can be observed [19].

A recent study also suggests that erythrocytes from PV patients are more adhesive since JAK2^V617F^ activates the erythrocyte adhesion receptor Lu/BCAM through an EPOR-independent RAP1/AKT signaling pathway (Figure 2) [20].

Finally, monocytes from MPN patients with JAK2^V617F^ have been found to have a defective negative regulation of toll-like receptor (TLR) signaling leading to increased production of the inflammatory cytokine TNF-α. These monocytes are insensitive to the anti-inflammatory cytokine IL-10, which in turn negatively regulates TNF-α production through TLR (Figure 2) [21]. Studies on TNF-α knockout mice have demonstrated that this cytokine is required for the development of an MPN-like disease [22]. Unrestrained production of TNF-α has been observed in an MPN patient but also in his identical twin, suggesting that it may be a genetic feature rather than a consequence of the disease [21]. In any case, the inflammatory environment can favor the maintenance and expansion of the JAK2^V617F^ mutant clone since these cells are resistant to inflammation whereas non-mutant cells are not [22]. Thus, the JAK2^V617F^ mutant clone seems to induce non-mutant cells to produce inflammatory cytokines, reinforcing the self-perpetuating environment for its continuous selection [23]. Finally, CD34+ progenitors of a PV patient with JAK2^V617F^ have been reported to use dual-specificity phosphatase 1 (DUSP1) to protect themselves against inflammatory stress and DNA damage, promoting their proliferation and survival in this microenvironment (Figure 2) [24].

### 3.2. CALR-Related Non-Canonical Signaling

**Figure 3:**
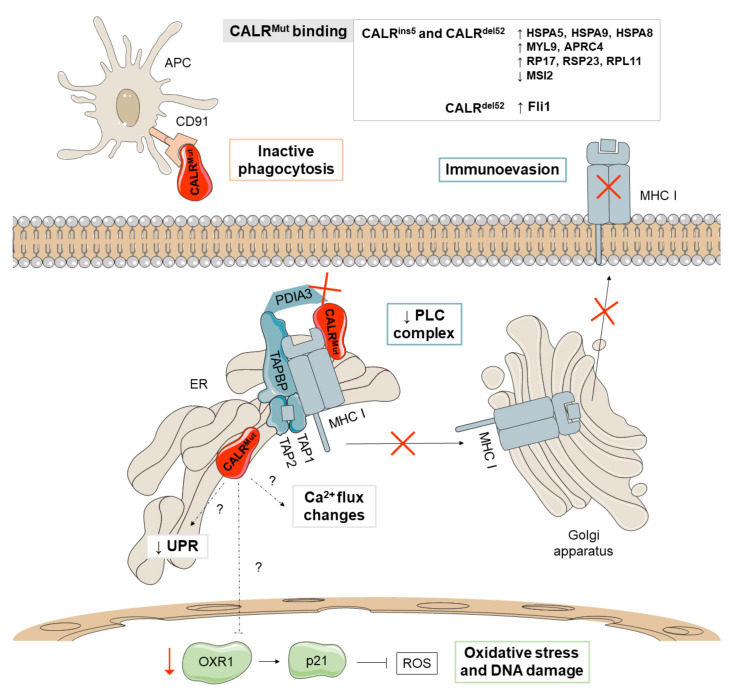
Major non-canonical mechanisms derived from CALR^Mut^. CALR^Mut^ (depicted in red) shows different binding affinities for proteins implicated in the unfolding protein response (UPR) (HSPA5, HSPA9, and HSPA8), proteins of the cytoskeleton (MYL9 and APRC4), and ribosomal proteins (RP17, RSP23, and RPL11), as well as reduced binding to MSI2, a transcriptional regulator that target genes mainly involved in cell cycle regulation. Additionally, CALR^Mut^ seems to reduce the activation of the pro-apoptotic pathway of the UPR and increases oxidative stress and DNA damage through the downmodulation of oxidation resistance 1 (OXR1). CALR^Mut^ also shows decreased binding affinities for PDIA3 and has a loss-of-function effect on the peptide loading complex (PLC), which mediates the loading of cellular antigens onto major histocompatibility complex class I (MHC-I) molecules, favoring immunoevasion. Mutations in *CALR* increase the secretion of the protein and bind to CALR receptors in antigen presenting cells (APCs), limiting their ability to phagocytize wild-type CALR-exposing cancer cells. The main differences between the phenotypes observed in patients with type 1 (del52) and type 2 (ins5) mutations have been attributed to thrombopoietin receptor (TPOR)-independent cytosolic calcium fluxes and the binding affinity for the transcription factor FLI1.

Several studies have identified novel mechanisms that collaborate with the activation of TPOR in CALR-mediated cellular transformation (Figure 3). CALR^Mut^ seems to cause reduced activation of the UPR pro-apoptotic pathway and to have an increased sensitivity to oxidative stress by the down-modulation of oxidation resistance 1 (OXR1) in K562 cells. These mechanisms lead to resistance to UPR-induced apoptosis and genomic instability, respectively [25]. Moreover, CALR^del52^ causes increased recruitment of the friend leukemia integration 1 (FLI1) transcription factor to the *MPL* promoter to enhance transcription [26], which suggests a promotion of tumorigenesis by modulating transcription through interactions with transcription factors in the nucleus.

Bioinformatic analyses of CALR^Mut^ revealed the appearance of potential phosphorylation sites for kinases that may have a role in the regulation of multiple cellular activities [27] and recent studies have shown that CALR^Mut^ causes increased binding affinities for proteins involved in the activation of the UPR (HSPA5, HSPA9, and HSPA8) and cytoskeletal (MYL9 and APRC4) and ribosomal proteins (RP17, RSP23, and RPL11), as well as reduced binding to MSI2, a transcriptional regulator that targets genes mainly involved in cell cycle regulation [26].

On the other hand, CALR is an integral part of the peptide loading complex (PLC), which mediates the loading of cellular antigens onto major histocompatibility complex class I (MHC-I) molecules. In addition to CALR, the PLC is composed of PDIA3, TAP-binding protein, TAP1, and TAP2. Specifically, CALR interacts with PDIA3 in a glycan-dependent manner and preserves steady-state levels of TAP-binding protein and MHC-I heavy chains. Besides, it rescues suboptimally assembled MHC-I molecules from post-ER compartments [28]. HEK293T cells lacking CALR expression show a reduction of properly loaded MHC-I on the cell surface, a defect that is not restored by expression of CALR^Mut^ [29]. Consistent with this, cells with CALR^Mut^ show reduced antigen presentation on MHC-I (Figure 3) [54] and decreased binding affinities for PDIA3 [26]. These results suggest that *CALR* mutations have a loss-of-function effect on PLC and, therefore, may contribute to the development of MPN by promoting immunoevasion after loss of tumor antigenicity [28]. Additionally, CALR operates as a key damage-associated molecular pattern (DAMP) when it is translocated to the outer cell membrane of dying cancer cells. CALR-exposing cancer cells deliver pro-phagocytic signals to antigen presenting cells (APCs) and activate dendritic cell efferocytosis. Mutations in *CALR* increase the secretion of the protein both in vitro and in vivo since the ER retention motif (KDEL) is compromised. Soluble CALR binds to CALR receptors in the APCs and limit their ability to phagocytise, leading to immunosuppressive effects (Figure 3) [30].

The wild-type CALR protein also regulates the activation of the stored-operated calcium entry (SOCE) machinery by interacting with PDIA3 and STIM1. Concretely, STIM1 is a protein of the SOCE machinery that leads to calcium mobilization. CALR^Mut^ has been shown to trigger TPOR-independent cytosolic calcium fluxes in megakaryocytes through defective interactions between CALR^Mut^, PDIA3 and the SOCE machinery. This results in uncontrolled proliferation of megakaryocytes that can be reversed with a SOCE inhibitor [31].

The type of *CALR* mutation has been associated with different disease features. Thus, type 1 mutations are more often associated with PMF (70%) or progression from ET to a myelofibrotic state [32], while type 2 mutations are more often associated with ET [91]. The mechanisms underlying this phenomenon have not been fully elucidated, but it has been demonstrated that type 2 mutants retain longer stretches of the negatively charged amino acids of wild-type CALR than type 2 mutants, which may neutralize the positive electronic charge generated at the C-terminal end. Additionally, type 1 mutant C-terminus generates greater changes in megakaryocyte cytosolic calcium flux than type 2 mutants [33].

### 3.3. Additional Non-Canonical Signaling

Non-canonical mechanisms affecting inflammatory signaling pathways and the bone marrow microenvironment have been widely observed in all MPNs, regardless of subtype and driver mutation.

#### 3.3.1. Inflammatory Signaling Pathways

**Figure 4:**
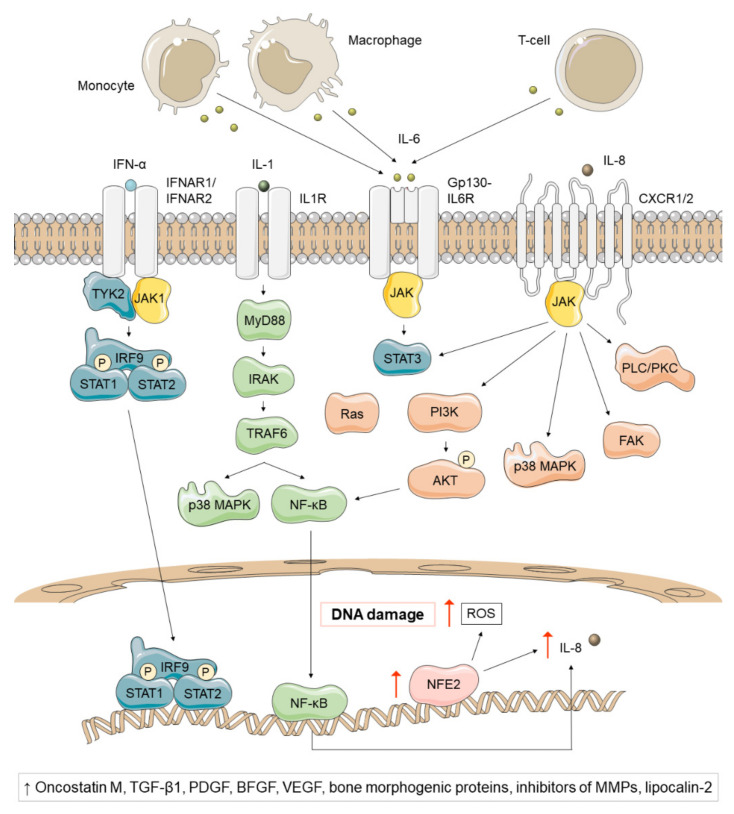
Non-canonical inflammatory signaling pathways affected in *Ph*-negative MPNs, regardless of subtype and driver mutation. The preleukemic niche of MPNs secretes high levels of IL-1, a proinflammatory cytokine that activates multiple downstream pathways, such as p38 MAPK and NF-κB. NF-κB, in turn, generates high levels of IL-8, a proinflammatory cytokine that binds to CXCR1 or CXCR2 and activates STAT3, PI3K/AKT, p38 MAPK, FAK and PLC/PKC. NFE2 overexpression has also been reported in most MPN patients and has been associated with high IL-8 levels and increased ROS and DNA damage. On the other hand, IL-6 is a proinflammatory cytokine produced by monocytes, macrophages and T-cells that signals via JAK1/STAT3, whose levels have been found elevated in JAK2^V617F^ PV and PMF patients. Finally, IFN-α is a key regulator of hematopoietic stem cells (HSCs) that depletes previously dormant hematopoietic stem progenitor cells (HSPCs) and enhances the immune response. A pathogenic role of oncostatin-M, TGF-β1, platelet-derived growth factor (PDGF), basic fibroblast growth factor (BFGF), VEGF, bone morphogenic proteins, inhibitors of matrix metalloproteinases (MMPs) and lipocalin-2 has been suggested in *Ph*-negative MPNs.

As previously noted, chronic inflammation is a characteristic feature of MPNs (Figure 4). In fact, MPN patients typically exhibit increased levels of inflammatory cytokines [34,106]. The impaired JAK2/STAT signaling is not the only contributor to inflammation in these diseases, as the inhibition of JAK2 is not sufficient to normalize the levels of inflammatory cytokines [35]. On the contrary, a significant enrichment of the NF-κB signaling pathway has been observed in both malignant and non-malignant cells in MPNs [36].

NFE2 overexpression has also been reported in most patients and seems to play a role in chronic inflammation [37,38]. NFE2 participates in inflammatory cascades by increasing IL-8 transcription and promotes proliferation by activating the expression of CDK4, CDK6 and cyclin D3 [39,40]. In addition, it produces reactive oxygen species (ROS), a group of highly reactive oxygen-containing molecules which participate in numerous biological processes [41]. This results in lipid and protein oxidation, increased oxidative DNA damage (8-oxo-G), and subsequent double-stranded DNA breaks that induce instability [38]. Excessive ROS production and subsequent oxidative stress confer a proliferative advantage to JAK2^V617F^ clones and activate proinflammatory pathways (NF-κB) that create more ROS. In this way, MPNs have recently been described as “a human inflammation model for cancer development”, as they are characterized by a self-perpetuating circle in which inflammation creates ROS which in turn creates more inflammation [42].

Multiple inflammatory signaling pathways such as IFN-α and IL-1β have been also found to be involved in the pathogenesis of MPN. Interferons are key regulators of HSCs. Data from murine PV JAK2^V617F^ models have shown that hematopoietic stem progenitor cells (HSPCs) become more proliferative and lose quiescence when treated with IFN-α, leading to their depletion [43,44]. The ability to deplete previously dormant malignant stem cells together with the enhancement of the immune response have made IFN-α one of the most efficient treatment options in MPNs [107]. On the other hand, IL-1β is a proinflammatory cytokine released by myeloid cells in response to TLR stimulation, that activates multiple downstream pathways such as NF-kB and p38 MAPK [45]. The preleukemic niche of MPNs secretes high levels of IL-1, which drives granulocyte/macrophage differentiation [46]. IL-6 and IL-8 also seem to participate in MPN pathogenesis. IL-6 is a proinflammatory cytokine produced by monocytes, macrophages and T-cells that signals via JAK1/STAT3 [45]. Several mouse models for MPNs have shown a high expression of IL-6 in both mutant and wild-type HSCs [23]. Additionally, elevated IL-6 levels have been observed in JAK2^V617F^ PV and PMF patients [47]. In fact, some studies point that IL-6 may participate in the progression of MPN to AML [45]. IL-8 is also a proinflammatory chemokine released in response to IL-1 or TNF-α that binds to CXCR1 or CXCR2 and activate JAK/STAT, PI3K/AKT, MAPK, PLC/PKC and FAK [45]. Elevated levels of IL-8 have been found in PV and ET patients [48].

Numerous cytokines have been implicated in mediating fibrosis, osteosclerosis and angiogenesis in PMF patients. Thus, several studies have suggested a pathogenic role for oncostatin-M [49], TGF-β1 [50,51], platelet-derived growth factor [51], basic fibroblast growth factor [50], VEGF [52], bone morphogenetic proteins [53], and inhibitors of matrix metalloproteinases [54,55].

Myeloid cells have been reported also to produce elevated levels of lipocalin-2 in PV, ET, and PMF patients. This protein increases the growth of bone marrow cells in PMF patients, but not in healthy donors. On the contrary, it increases reactive oxygen species, DNA damage, and apoptosis in normal cells, but not in PMF patients. Lipocalin-2 also induces the expression of factors that contribute to fibrosis, such as VEGF, TGF-β1, bone morphogenetic protein-2, RUNX2, osteoprotegerin and collagen type I [56,108].

Heat shock proteins (HSPs) are key players during inflammation. HSP90 stabilizes numerous proteins, such as JAK2. The HSP70 family is composed of some proteins (HSPA5, HSPA8, and HSPA8) that have been found to be enriched in fractions bound to CALR^Mut^ [26]. HSP70 also seems to contribute to cell proliferation through regulation of JAK2/STAT signaling. In fact, the inhibition of HSP70 expression in an ex vivo model of PV and ET increased apoptosis of the erythroid lineage and decreased JAK2 signaling [57]. HSP70 also activates TLR2 and TLR4, leading to NF-κB activation, rapid calcium flux, and TNF-α, IL1-β and IL-6 production [58]. Moreover, HSP70 can be secreted as a “danger signal” and bind peptides to form a complex that binds to cell surface receptors, such as CD91 and Lox-1 [59].

Finally, there is also evidence for a link between inflammation and thrombosis. Thrombosis in MPN patients has been associated with an increased platelet-leukocyte interaction. While MPN leukocytes overexpress the surface protein CD11b, its receptor (CD62p) is upregulated on platelets. This results in increased formation of leukocyte-platelet complexes [60,61,62].

#### 3.3.2. Bone Marrow Microenvironment

**Figure 5:**
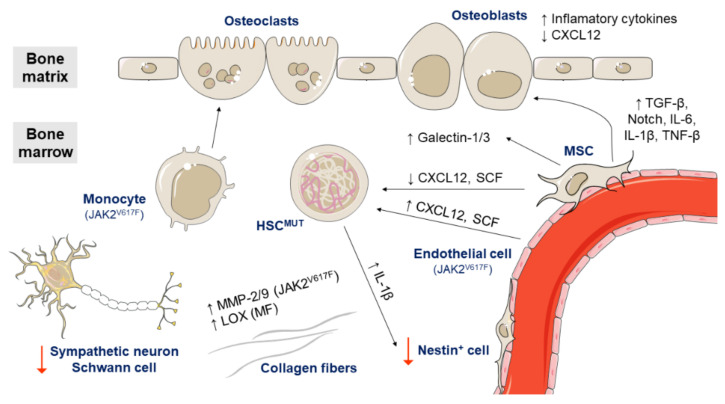
Role of the bone marrow microenvironment in the pathogenesis of *Ph*-negative MPNs. Endothelial cells expressing JAK2^V617F^ increase the expression of CXCL12 and stem cell factor (SCF) and cause the expansion of HSCs and progenitor cells. On the other hand, mesenchymal stem cells (MSCs) negative for JAK2^V617F^ show a reduced expression of CXCL12 and SCF. MSCs also overexpress galectin-1 in all MPN subtypes and galectin-3 in PV patients, and promote the expansion of osteoblasts by cell contact and excessive TGF-1β, Notch, IL-6, IL-1β, and TNF-β signaling. Osteoblasts overproduce inflammatory cytokines and reduce CXCL12 expression. By contrast, monocytes with JAK2^V617F^ seem to increase osteoclast forming ability and favor the survival of clonal HSCs. Meanwhile, clonal HSCs produce high levels of IL-1β, which induces nestin-positive MSCs death. Additionally, the sympathetic nerve fibers supporting Schwann cells are reduced in the bone marrow of MPN patients. Regarding the extracellular matrix, MPN patients with JAK2^V617F^ show increased levels of MMP-2 and MMP-9 and patients with primary myelofibrosis (PMF) have increased levels of all LOX family members.

The bone marrow microenvironment is a complex and dynamic structure composed of multiple cell types. Clonal HSCs in MPNs interact with other cells in this microenvironment and remodel it allowing further malignant expansion (Figure 5). There is a growing evidence that endothelial cells, mesenchymal stem cells, stromal cells, osteoblasts, and osteoclasts may contribute to the pathogenesis of these diseases in the bone marrow [63].

In a mouse model, endothelial cells expressing JAK2^V617F^ have been shown to be capable of causing the expansion of hematopoietic stem and progenitor cells, which could be caused by increased expression of the cytokines CXCL12 (C-X-C motif chemokine ligand 12) and SCF (stem cell factor) by endothelial cells [64,65].

Mesenchymal stem cells (MSCs) also seem to be important in the pathogenesis of MPNs. In contrast to endothelial cells expressing JAK2^V617F^, MSCs negative for JAK2^V617F^ have been reported to reduce the expression of CXCL12 and SCF [109,110]. They also support HPSC proliferation [66] and overexpress galectin-1 in all MPN subtypes and galectin-3 in PV patients [67]. Galectins mediate cell adhesion and stimulate cell migration, proliferation and apoptosis through interactions with integrins, laminin and fibronectin. In addition, MSCs promote the expansion of osteoblasts by cell contact and excessive TGF-β1, Notch, IL-6, IL-1β, and TNF-β signaling. Abnormal osteoblasts overproduce inflammatory cytokines, promote fibrogenesis and reduce CXCL12 expression [88]. By contrast, monocytes with JAK2^V617F^ seem to increase osteoclast forming ability in MPN patients, favoring the survival of clonal HSCs [68].

A recent study has recently found numerous differences between the bone marrow niche of ET and PV patients. In ET, the HSPCs move faster and more frequently towards the endosteal niche and the number of osteoblasts and osteoclasts increases. However, in PV, only the non-endosteal sinusoids are dilated [69]. Other studies have demonstrated that the sympathetic nervous system has a role in the bone marrow niche of MPN patients. Specifically, sympathetic nerve fibers supporting Schwann cells and nestin-positive MSCs are reduced in the bone marrow of MPN patients. In a murine MPN model harboring JAK2^V617F^, stem cells secreted IL-1β, which induces nestin-positive MSCs death and enables disease expansion [70].

Regarding the extracellular matrix, several studies have also pointed to a role of matrix metalloproteinases (MMPs) and lysyl oxidase (LOX) in the pathogenesis of MPNs. MPN patients with JAK2^V617F^ show increased levels of MMP-2 and MMP-9 [71] and patients with PMF have increased levels of all LOX family members. LOX is involved in collagen cross-linking and promotes fibrogenesis [72].

## 4. Disease Modifiers

**Figure 6:**
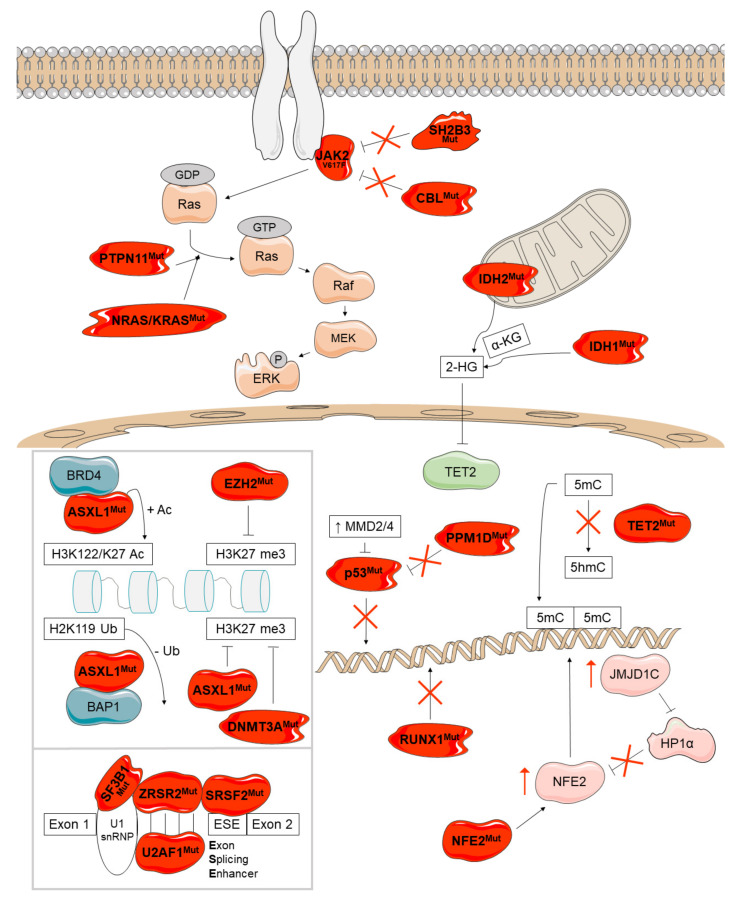
Overview of the disease-modifying genes mutated in *Ph*-negative MPNs and their molecular consequences. These mutations occur in genes affecting epigenetic regulation (*ASXL1*, *EZH2*, *DNMT3A*, *TET2*, *IDH1*, and *IDH2*), tumor suppression (*TP53* and *PPM1D*), transcription regulation (*RUNX1* and *NFE2*), splicing (*SRSF2, U2AF1, SF3B1*, and *ZRSR2*), and other signaling pathways (*SH2B3*, *CBL*, *NRAS*/*KRAS*, *PTPN11*). Mutant proteins are depicted in red.

Several non-driver somatic mutations have been identified in MPN patients. According to recent studies, more than 80% of patients with PMF [73] and over 50% of PV/ET patients have at least one additional somatic mutation of this type [74]. These mutations occur in genes affecting a wide variety of processes like epigenetic regulation, tumor suppression, transcription regulation or splicing, but also additional signaling pathways (Figure 6). They often modify the course of the disease and the presence of more than one such aberration has been associated with a worse survival [75]. 

### 4.1. Epigenetic Regulation

The most common non-driver somatic mutations affect epigenetic regulation and have been identified in *ASXL1* (ASXL transcriptional regulator 1), *EZH2* (enhancer of zeste polycomb repressive complex 2 subunit), *DNMT3A* (DNA methyltransferase 3 alpha), *TET2* (TET methylcitosine dioxygenase 2), *IDH1* and *IDH2* (isocitrate dehydrogenase NADP+, 1 and 2).

The products of *ASXL1* and *EZH2* are involved in chromatin modification (Figure 6, upper box). Normal ASXL1 interacts with the polycomb repressor complex 2 (PRC2) and enhances its function as methylator of H3K27. H3K27 trimethylation results in the silencing of the *HOXA* gene family which participates in chromatin remodeling. Additionally, ASXL1 interacts with BRCA1-associated protein 1 (BAP1), creating the polycomb group repressive deubiquitinase complex, which globally removes monoubiquitin from H2AK119 and locally at *HOXA* and *IRF8* in HSCs [76,111,112]. *ASXL1* mutations are almost exclusively frameshift and nonsense mutations in exon 12, decrease H3K27 trimethylation [111] and enhance the activity of the ASXL1-BAP deubiquitinase complex [113]. This causes the deregulated expression of genes critical for HSC self-renewal and differentiation, as well as more open chromatin in c-Kit+ cells. Mutant ASXL1 also binds to the bromodomain-containing protein 4 (BDR4), resulting in the phosphorylation of RNA polymerase II and the acetylation of H3K27 and H3K122, which lead to the upregulation of genes governing myeloid differentiation [76,114]. Another mechanism reported for mutant ASXL1 consists of the repression of TGF-β pathway through H3K and H4K deacetylation [115]. Although normal ASXL1 activates the retinoic acid receptor [116] and interacts with the peroxisome proliferator activated receptor gamma (PPARγ) to repress lipogenesis [117], the effects of *ASXL1* mutations on these mechanisms are still unknown. In summary, the consequences of *ASXL1* mutations are diverse and are not fully elucidated; the mutant protein shows a loss of function in some mechanisms but a gain of function in others. 

*EZH2* encodes a histone lysine N-methyltransferase that constitutes the catalytic component of PRC2. The majority of *EZH2* mutations are missense with loss of function effects resulting in the silencing of *HOXA9.* This supports myeloid progenitor self-renewal and leukemic transformation [118,119].

*DNMT3A*, *TET2*, *IDH1* and *IDH2* encode DNA methylation modifiers (Figure 6). *DNMT3A* encodes a *de novo* DNA methyltransferase responsible for DNA methylation at CpG dinucleotides. The mutation most frequently observed is p.R882H, that impairs the CpG specificity, flanking sequence preference and DNMT3A enzymatic activity [120]. Mechanistic studies in mice indicate that mutant DNMT3A decreases PRC2 recruitment at H3K27 favoring accessibility at enhancer chromatin marks and persistent HSC gene expression. JAK2^V617F^ patients also harboring *DNMT3A* mutations show aberrant self-renewal and altered inflammatory signaling pathways [121]. 

*TET2* encodes an enzyme that catalyzes the oxidation of 5-methylcytosine (5mC) to 5-hydroxymethylcytosine (5-hmC). Mutations in *TET2* are nonsense or missense changes that lead to a loss of function [122] and DNA hypermethylation due to decreased production of 5-hmC. Mutant TET2 increases the expression of HSC self-renewal genes and sensitizes hematopoietic cells to acquire other mutations and leads to significant myeloid lineage skewing [123] and increased IL-6 production [124]. The order of mutation acquisition can influence the MPN phenotype; mutations in *TET2* arising prior to JAK2^V617F^ favors the ET phenotype, but the acquisition of JAK2^V617F^ in a *TET2* non-mutated background is more likely to result in the PV phenotype [125]. 

*IDH1* and *IDH2* encode isocitrate dehydrogenases that catalyze decarboxylation of isocitrate into alpha ketoglutarate (α-KG). While IDH1 acts in the cytosol, IDH2 works in the mitochondria. The most common *IDH1* (p.R132H and p.R132C) and *IDH2* mutations (p.R140Q) increase 2-hydroxyglutarate (2-HG) production. 2-HG prevents histone demethylation and the expression of lineage-specific differentiation genes, leading to a block to cell differentiation [126,127,128]. This compound can also bind ten-eleven translocation (TET) and Jumonji proteins, inhibiting their functions [129]. IDH mutations have also been associated to enhanced aberrant splicing of mutant SRSF2, leading to genomic instability and risk of leukemic transformation [130].

### 4.2. Tumor Suppression

*TP53* (tumor protein P53) and *PPM1D* (protein phosphatase, Mg^2+^/Mn^2+^ dependent 1D or P53-induced protein phosphatase 1) are involved in tumor suppression (Figure 6). TP53 is a transcription factor that responds to DNA damage inducing transcriptional programs that result in cell cycle arrest or apoptosis [131]. *TP53* mutations are missense changes with several non-mutually exclusive effects: loss of function, gain of function, and dominant-negative effect on normal TP53 [77]. It has been also demonstrated that mutant TP53 increases HSC self-renewal and resistance to cellular stress [132]. There are several upstream regulators of TP53, which are overexpressed in MPNs, such as MDM2 and MDM4. Both of them inhibit TP53 function by facilitating nuclear export and by inducing its degradation [133]. 

PPM1D is a serin-threonine phosphatase which negatively regulates TP53 and is transcriptionally upregulated on TP53 induction [134]. Mutations in *PPM1D* are truncating and frameshift changes in exon 6 that lead to a protein that lacks a carboxyterminal degradation domain. This results in altered cell cycle progression, decreased apoptosis and reduced mitochondrial priming [135].

### 4.3. Regulation of Transcription

*RUNX1* (RUNX family transcription factor 1) and *NFE2* (nuclear factor, erythroid 2) encode transcription factors and have been also found mutated in MPNs (Figure 6). RUNX1 contains a runt homology domain (RHD) responsible for DNA binding and heterodimerization with core binding factor β (CBF-β). Through this interaction, RUNX1 controls key hematopoietic transcriptional programs. Specifically, RUNX1 participates in hematopoietic differentiation, cell cycle regulation, ribosome biogenesis, and p53 and TGF-β pathways [136]. RUNX1 mutations are missense, frameshift, and non-sense changes that inactivate the protein leading to a reduced myeloid differentiation and an increase in HSC self-renewal [77].

Mutations described in *NFE2* are a 4-amino acid in-frame deletion and frameshift changes that lead to a carboxy-terminally truncated protein [40]. Mutant NFE2 promotes myelopoiesis and causes elevated expression of wild-type NFE2 and histone demethylase JMJD1C maybe by a decreased binding of the repressor HP1α [137].

### 4.4. Splicing

Pre-mRNA splicing is catalyzed by the spliceosome, a complex of five snRNPs and multiple proteins. Mutually exclusive mutations in RNA splicing factors encoded by *SRSF2* (serine and arginine rich splicing factor 2), *U2AF1* (U2 small nuclear RNA auxiliary factor 1), *SF3B1* (splicing factor 3b subunit 1) and *ZRSR2* (zinc finger CCCH-type, RNA binding motif and serine/arginine rich 2) have been reported in MPNs (Figure 6, lower box).

SRSF2 contains a ribonucleoprotein with an RNA binding motif and a carboxyl-terminal serine/arginine rich domain [138], both involved in the recognition and binding to the RNA sequences GGNG and CCNG in exon splicing enhancers (ESEs). The most frequent mutation is p.P95H, that leads to a preferential recognition of CCNG motifs and alters the balance of splicing of multiple pre-mRNAs, which cause downregulation of *EZH2* [139], as well as the mis-splicing of *CASP8,* which activates NF-κB signaling [140]. The expression of mutant SRSF2 has also been demonstrated to cause accumulation of R loops, replication stress and activation of ATR-Chk1 signaling [141,142]. Additionally, mutant SRSF2 seems to predominantly form RUNX1a over RUNX1b and regulate DNA stability [143,144].

U2AF1 recognizes pyrimidine-rich tracts with a conserved terminal AG in 3**′** splice sites [145]. The most prevalent somatic mutations affect Q157 and its surroundings; p.Q157 mutants generate mis-splicing of ARID2 and EZH2 [123] and are associated with a worse outcome [146]. Patients can also harbor mutations in serine 34 (p.S34F/Y) that cause different expression and splicing patterns than p.Q157 mutations and have been associated with increased splicing, accumulation of R loops and exon skipping [142,147]. Both types of mutations are located within the CCCH zinc fingers of U2AF1, that are critical for RNA binding [148]. This protein has also been shown to bind to mRNA and repress translation; p.S34F mutation seems to affect the translation of hundreds of mRNAs, but the effect of the other mutations on translation is still unknown [149]. 

ZRSR2 heterodimerizes with U2AF2 and participates in the recognition of the 3**′** splice site. Mutations in this gene are mostly frameshift and nonsense loss-of-function changes that affect splicing and lead to intron retention. Mutant ZRSR2 has been reported to cause increased MAPK and ERBB signaling in myelodysplastic syndromes [150]. 

SF3B1 forms part of the spliceosome complex. Mutations in *SF3B1* are missense changes (p.K700E and p.H662Q) that cause alternative 3**′** splice site selection [151]. These mutations block erythroid maturation [152] and modify the expression of genes involved in RNA processing, cell cycle, heme metabolism and nonsense-mediated RNA decay [77].

### 4.5. Additional Signaling Pathways

Finally, other mutations have been found in *SH2B3* (SH2B adaptor protein 3, previously known as *LNK*), *CBL* (CBL proto-oncogene), *NRAS* and *KRAS* (NRAS and KRAS proto-oncogene, GTPase) and *PTPN11* (protein tyrosine phosphatase non-receptor type 11), all of them encoding elements involved in signaling (Figure 6).

SH2B3 is an adaptor protein that interacts with and inhibits signaling through cytokine receptors and kinases such JAK2 [153,154,155] decreasing the proliferation of hematopoietic cells [156,157,158]. In addition, this protein can recruit the E3-ubiquitin ligase CBL for degradation of receptors and other molecules [157]. Mutations in *SH2B3* are mainly missense changes that disrupt the negative-feedback loops on growth stimulation [155,157]. 

CBL recognizes and ubiquitinates activated tyrosine kinase receptors and JAK2 leading to their proteasomal degradation. Mutations in *CBL* are mostly missense changes that reduce the E3 ligase activity and the degradation of its substrates [159,160,161]. However, they are not merely loss-of-function mutations since *CBL* knockout cells show increased cytokine sensitivity [162]. 

Missense substitutions affecting *NRAS*/*KRAS* favor the GTP-bound state of RAS, causing a constitutive activation of growth signaling [163].

Finally, *PTPN11* encodes a protein tyrosine phosphatase which dephosphorylates RAS [164]. *PTPN11* mutations increase its phosphatase activity [165], leading to a high dephosphorylation of RAS which increases the activation of RAS-RAF-MEK-ERK pathway [166].

## 5. Additional Factors Involved in Disease

There are several factors that have been shown to influence heterogeneity in MPN phenotypes, such as the HSC in which the mutation appears first, genetic background, gender, age, and environmental factors.

HSCs are highly heterogeneous and carry a lineage-bias [167]. It has been demonstrated that the acquisition of a driver mutation in a platelet-biased HSC may drive to an ET phenotype, whereas the PV phenotype is more probable when mutation is acquired in balanced/myeloid-biased HSCs [168].

It is well known that there is an association between the *JAK2* haplotype 46/1 or GGCC and MPNs. This haplotype is found in 24% of the population and in the 56% of MPN patients [169] increasing the susceptibility of developing a *JAK2* mutation, but also to *CALR* mutations and weakly to *MPL* mutations [169,170]. Recent studies have identified several SNPs in different loci which have been associated with an increased risk of developing some MPN subtypes [171,172,173].

Regarding gender, the ET phenotype has been mostly reported in women, while PV/PMF are more prevalent in men [174,175]. Women seem to have a greater symptom burden than men [175], but the male gender has been associated with a higher likelihood of myelofibrotic transformation in ET patients [176].

The incidence of MPNs also increases with age, and this factor is the strongest predictor of death in PV and ET [177,178]. This phenomenon has been related to the influence of aging in hematopoiesis, maybe due to a greater probability of acquiring somatic mutations in HSCs [78] favored by a pro-inflammatory state due to the accumulation of inflammatory cytokines associated with age [45]. This higher probability would also explain the increased risk of disease progression in these patients [78].

Retrospective observational studies have reported that the occupational exposure to benzene and/or petroleum, prior blood donation (specifically for PV) [179], and smoking [180,181] are associated with a higher risk of MPNs.

## 6. Conclusions

The understanding of the pathogenesis of MPNs has undergone a complete revolution in the last 15 years, especially since the p.V617F mutation in *JAK2* was characterized. Since then, MPNs have basically been considered signaling disorders, especially affecting the JAK2/STAT pathway, but also the MAPK/ERK and PI3K/AKT pathways. Further characterization of mutations in *MPL*, and the mechanism by which *CALR* mutations activate TPOR, reinforced this view. However, although the pathogenic mechanisms of the JAK2, TPOR, and CALR mutants seem quite straightforward and simple, various studies have shown that these alterations can cause more complex disturbances in the cell through non-canonical mechanisms. This, together with the characterization of new somatic genetic alterations that affect genes involved in other processes and signaling pathways, have revealed the complexity of the pathogenesis of MPN, which could partly explain the phenotypic heterogeneity observed among patients.
